# A single point mutation in *Ms44* results in dominant male sterility and improves nitrogen use efficiency in maize

**DOI:** 10.1111/pbi.12689

**Published:** 2017-02-07

**Authors:** Tim Fox, Jason DeBruin, Kristin Haug Collet, Mary Trimnell, Joshua Clapp, April Leonard, Bailin Li, Eric Scolaro, Sarah Collinson, Kimberly Glassman, Michael Miller, Jeff Schussler, Dennis Dolan, Lu Liu, Carla Gho, Marc Albertsen, Dale Loussaert, Bo Shen

**Affiliations:** ^1^ DuPont Pioneer Johnston IA USA

**Keywords:** *Ms44*, lipid transfer protein, male sterility, grain yield, NUE, maize

## Abstract

Application of nitrogen fertilizer in the past 50 years has resulted in significant increases in crop yields. However, loss of nitrogen from crop fields has been associated with negative impacts on the environment. Developing maize hybrids with improved nitrogen use efficiency is a cost‐effective strategy for increasing yield sustainably. We report that a dominant male‐sterile mutant *Ms44* encodes a lipid transfer protein which is expressed specifically in the tapetum. A single amino acid change from alanine to threonine at the signal peptide cleavage site of the Ms44 protein abolished protein processing and impeded the secretion of protein from tapetal cells into the locule, resulting in dominant male sterility. While the total nitrogen (N) content in plants was not changed, *Ms44* male‐sterile plants reduced tassel growth and improved ear growth by partitioning more nitrogen to the ear, resulting in a 9.6% increase in kernel number. Hybrids carrying the *Ms44* allele demonstrated a 4%–8.5% yield advantage when N is limiting, 1.7% yield advantage under drought and 0.9% yield advantage under optimal growth conditions relative to the yield of wild type. Furthermore, we have developed an *Ms44* maintainer line for fertility restoration, male‐sterile inbred seed increase and hybrid seed production. This study reveals that protein secretion from the tapetum into the locule is critical for pollen development and demonstrates that a reduction in competition between tassel and ear by male sterility improves grain yield under low‐nitrogen conditions in maize.

## Introduction

Nitrogen (N) is an essential nutrient for plant growth and development and is an important factor determining maize grain yield. Unfortunately, only about 1/3 of applied nitrogen is utilized by crops, while 2/3 is lost to the environment (McAllister *et al*., 2012; Raun and Johnson, 1999; Xu *et al*., 2012). Excessive application of nitrogen fertilizer is associated with negative impacts on the environment including eutrophication of lakes and rivers, and toxic levels of nitrates in underground water for human consumption (Good and Beatty, 2011; Liu *et al*., 2013). In addition, nitrogen fertilizers account for ~25% of the total input costs (seeds, fertilizers and pesticides) in maize production. Improving nitrogen use efficiency (NUE) through genetic engineering in major crops is still in its infancy and far from ready for commercialization. Multiple studies have reported an improvement in NUE in *Arabidopsis*, rice, wheat and maize using transgenic approaches, but the NUE of the transgenic lines has not been validated in field trials using elite germplasm (McAllister *et al*., 2012; Xu *et al*., 2012). On the other hand, through plant breeding efforts, newer maize hybrids developed for North America have shown significant improvements in NUE compared to older hybrids. Grain yields have increased steadily in the United States in the past 30 years, while N fertilizer application rate has not been increased significantly (Ciampitti and Vyn, 2014; Han *et al*., 2015). Further improvements in maize NUE will not only reduce the environmental footprint of nitrogen fertilizer and cost to the grower, but also increase grain yields at current N application rates. In the majority of African countries, improving NUE is critical for food security as farmers cannot afford to apply more N fertilizer.

Maize is a monoecious plant with separate male and female flowers on the same plant. To produce pure hybrid seed, detasseling of the female inbred parent is employed widely by the seed industry. Although mechanical detasseling is effective in maize hybrid seed production, it is time‐consuming and labour‐intensive. In addition, damage to the top leaves during detasseling reduces hybrid seed yield. Male sterility is the most efficient way to ensure cross‐pollination (Chen and Liu, 2014; Kempe and Gils, 2011; Perez‐Prat and van Lookeren Campagne, 2002). Cytoplasmic male sterility (CMS) has been used to produce hybrid seed in both maize and rice. CMS is based on mutations in mitochondrial DNA, and male fertility can be restored by specific nuclear restorer genes. However, CMS is difficult to develop as it may not work across germplasms and sterility and fertility restoration may not be stable in certain environments. A CMS system requires a male‐sterile line, a maintainer line and a fertility restoration line, which involves a complex integration in the breeding process (Chen and Liu, 2014; Weider *et al*., 2009). Alternatively, nuclear genetic male sterility is stable in different germplasms and growth environments. Over 40 genetic male‐sterile mutants in maize have been identified and characterized with the majority of the mutations being recessive (Skibbe and Schnable, 2005). However, it is problematic to use genetic male‐sterile mutants in hybrid seed production because the male‐sterile female inbred cannot be self‐pollinated. Recently, *ms45*, a recessive genetic male‐sterile mutant, was cloned and a hybrid seed production technology (SPT) was developed (Wu *et al*., 2015). The SPT construct contained a wild‐type *Ms45* gene for fertility restoration, an α‐amylase gene to disrupt pollination and a seed colour marker for seed sorting. The maintainer line containing the SPT construct was fertile and shed 50% nontransgenic *ms45* pollen and 50% transgenic *ms45* pollen. Pollen harbouring the SPT transgene were unable to germinate due to depletion of starch by α‐amylase, whereas pollen that did not carry the SPT transgene were able to fertilize homozygous mutant plants. The resulting homozygous *ms45* seeds were nontransgenic, with respect to the SPT transgene, and could be used for hybrid seed production. Other transgenic male sterility systems have also been reported, such as the split‐gene system for hybrid wheat (Kempe *et al*., 2014) and the SeedLink for rapeseed from Bayer Crop Science (Newhouse *et al*., 1996).

During the early phases of reproductive growth (initiation of the ear shoot and tassel) in maize, tassel development outcompetes ear development for nitrogen and other nutrients, especially under stress conditions when resources are limited. We hypothesize that a reduction in tassel apical dominance through male sterility may promote greater ear development at anthesis and improve grain yield under stress conditions. *Ms44* is a dominant male‐sterile mutant identified from an EMS mutagenized population (Albertsen and Trimnell, 1992) and can be used easily to produce male‐sterile hybrids. In this study, we cloned the *Ms44* gene which encodes a putative lipid transfer protein expressed specifically in the tapetum. A single amino acid change from alanine to threonine at the predicted secretory signal sequence cleavage site was responsible for dominant male sterility. Expression of an artificial microRNA targeting and silencing the *Ms44* gene restored male fertility fully in an *Ms44* mutant background. *Ms44* male‐sterile plants showed a reduced tassel growth and enhanced ear growth, resulting in an average of 9.6% increase in kernel number per ear. Hybrids carrying the *Ms44* allele showed a 4%–8.5% yield increase under N‐limited conditions. These findings indicated that protein secretion from tapetal cells into the locule was essential for proper pollen development and that a reduction in competition between tassel and ear by male sterility improved grain yield under low‐N conditions.

## Results

### *Ms44* map‐based cloning and characterization

**Figure 1:**
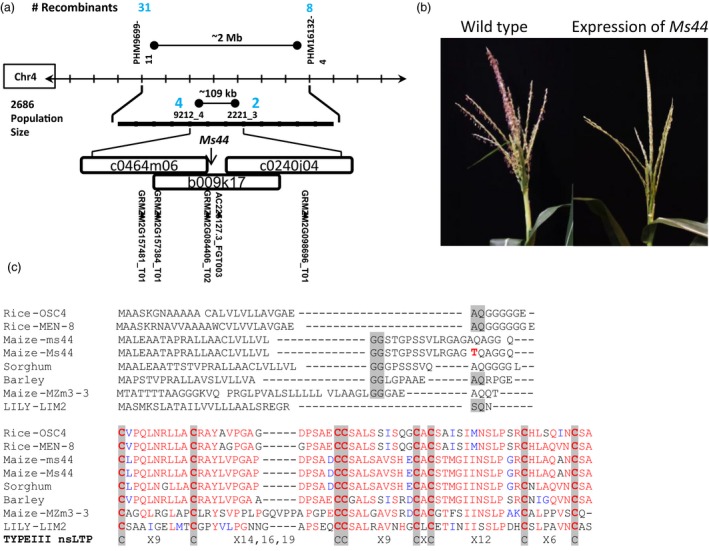
*Ms44* map‐based cloning. (a) Fine mapping of *Ms44*. (b) Expression of *Ms44* genomic in wild‐type maize. *Ms44* genomic fragment was isolated from *Ms44* mutant and introduced into wild type. Wild‐type plant with expression of *Ms44* dominant allele showed male‐sterile phenotype. (c) Protein sequence alignment of Ms44 with other plant LTPs. The N‐terminal sequence up to the first conserved cysteine is hand‐aligned. SSS sites predicted by SignalP are shaded grey. The barley sequence had two highly predicted SSS sites. The threonine mutation in Ms44 is red bold. The remaining protein is a CLUSTALW alignment. Conserved cysteine residues are shaded bold and compared to consensus type III nsLTPs. The accession numbers for the LTPs are as follows: LILY‐LIM2 (Q43534); Sorghum (XP_002445754); Barley (BAK05897); Rice‐OSC4 (BAD09233); Rice‐MEN‐8 (XP_006660357); and Maize‐MZm3‐3 (NP_001105123).

*Ms44* was previously mapped to chromosome 4 (Albertsen and Trimnell, 1992). To understand the molecular basis of dominant male sterility, *Ms44* was isolated by map‐based cloning (Figure 1a). In a large population of 2686 individuals, *Ms44* was validated as mapping to chromosome 4 and narrowed down to a 109‐kb region between markers 9212_4 and 2221_3. There were five annotated genes in this interval, and AC225127.3_FGT003 was selected as the apparent candidate based on its male preferred expression pattern (Wright *et al*., 1993). Sequence analysis of AC225127.3_FGT003 from the *Ms44* mutant revealed a single base change in the gene as compared to its original W23 parent. To verify that this single base pair change was the causal *Ms44* mutation, the allele was transformed into wild‐type maize. Expression of the candidate Ms44 genomic clone that included 1.2 kb upstream and 0.8 kb downstream from the coding sequence resulted in a male‐sterile phenotype (Figure 1b), confirming that AC225127.3_FGT003 was the *Ms44* gene.

The *ms44* coding region is comprised of two exons and contains a small 101‐bp intron. The first exon of *ms44* encodes all but the last two amino acids of the predicted protein. The *Ms44* encodes a lipid transfer protein containing a domain found in the superfamily of bifunctional inhibitor/plant lipid transfer protein/seed storage helical domain proteins. The *Ms44* protein contains eight conserved cysteine residues, important for secondary structure, and belongs to the type III or type C nonspecific lipid transfer protein (nsLTP), based on sequence analysis (Figure 1c). Homology searches with the *Ms44* protein revealed a large number of male‐expressed plant‐specific proteins, all of which have homology to ns‐LT proteins. Shown in Figure 1c is an alignment of the N‐terminal region of these proteins which show a higher variability than the C‐terminal region, but reveal that all contain a predicted secretory signal sequence (SSS), which is predicted to be processed in the endoplasmic reticulum (ER) for extracellular secretion (Von Heijne, 1986).

### Spatiotemporal expression and protein localization of *Ms44*

*Ms44* is an anther‐specific gene. Expression was first detected during meiosis and persists through the quartet and uninucleate stages of microspore development, but was not found after the first pollen mitosis (Figure S1a–b). To assess spatial expression of the *ms44* gene, transgenic constructs were made using the *ms44* promoter driving the expression of the fluorescent protein Zs‐Green. As shown in Figure S1c, fluorescence was detected only in the tapetal cell layer of the anther. The signal was detectable but weak during meiosis and increased during quartet and microspore release stages of development, consistent with the temporal expression data.

The SNP found in the *Ms44* allele (G to A) causes an amino acid change from an alanine to a threonine at amino acid 37. The mutation is 14 amino acids downstream of the predicted SSS cleavage site, which is predicted to cleave between G23 and G24. However, comparing predicted SSS sites between the other male specific nsLTPs in Figure 1c reveals a discrepancy between the predicted sites. Half of the compared proteins have a predicted SSS cleavage site equivalent to the G23/G24 position, while the others are predicted to cleave at the comparable A37/Q38 position. Notably, the barley protein had roughly equivalent SSS scores at both positions. Additionally, the comparable A37/Q38 residues are conserved in these sequences except for the Lily sequence which has a S/Q, whereas the G/G site is not conserved. Therefore, there is a question as to where SSS cleavage occurs in Ms44, and whether the mutation from an alanine to a threonine at amino acid 37 affects this process. To investigate whether the A37/Q38 are critical for signal sequence cleavage, point mutations were made around the A37 amino acid. If cleavage occurs between A37 and Q38, A37 can be considered to be at the −1 position, with the Q38 being at the +1 position or the first amino acid in the mature form of the protein. Amino acid changes were made at both the −1 and +1 positions and were chosen using consensus amino acid patterns near signal sequence cleavage sites (Von Heijne, 1986). At the −1 position, variant clones were made having A37G or A37V, with a single variant at the +1 position, Q38P. The A37G was the only variant that would be predicted to cleave.

**Figure 2:**
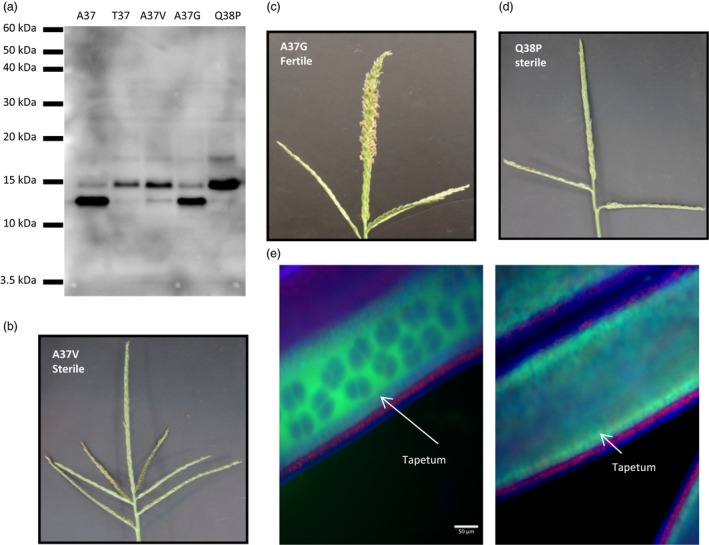
Disruption of signal peptide processing by *Ms44* mutation. (a) *In vitro* protein processing of Ms44 variants. (b) Transgenic tassel of Ms44 A37‐to‐V37 change. (c) Transgenic tassel of Ms44 A37‐to‐G37 change. (d) Transgenic tassel of Ms44 Q38‐to‐P38 change. (e) Localization of MS44::AcGFP fusion proteins in anthers at dyad‐to‐tetrad stages of microspore development by wide‐field fluorescence microscopy. Wild‐type ms44::AcGFP fusion (left) and mutant Ms44::AcGFP fusion (right) were expressed under the *ms44* promoter. Red signal indicates autofluorescence of chloroplasts in the endothecium. Tapetum cell layer is indicated by arrow.

A eukaryotic cell‐free *in vitro* synthesis system containing the machinery required for signal peptide processing was employed to measure the effect of *Ms44* amino acid changes on protein processing. As shown on the protein blot in Figure 2a, only the wild‐type A37 and the A37G variant had the majority of the detectable *Ms44* protein processed to a lower molecular weight form. The A37T, A37V and Q38P showed little to no protein processing, having the majority of *Ms44* protein uncleaved. This indicates that A37 and Q38 are critical for signal peptide cleavage and confirms that the T37 found in the dominant *Ms44* mutant abolishes this processing. Ultimately to demonstrate that a lack of SSS processing in the *Ms44* protein is responsible for the dominant male sterility phenotype, we used the amino acid variant clones of *ms44* described above and transformed them into maize plants. Single‐copy plants were selected and grown to maturity where male fertility phenotypes were evaluated. As shown in Figure 2b–d, transgenic plants containing cleavable A37G variant were male fertile, while variants that disrupt *Ms44* signal peptide cleavage, A37V and Q38P, were completely male‐sterile. These results confirm that a lack of *Ms44* signal peptide cleavage results in a disruption of pollen development leading to dominant male sterility in maize.

To confirm that *Ms44* mutation affects protein processing and secretion, both wild‐type and mutant *Ms44* genes were fused with AcGFP, and separately introduced into plants to assess spatial localization of the two Ms44 protein forms. Anthers were harvested for analysis using both light and confocal microscopy. As shown in Figure 2e, the Ms44 mutant protein fusion was confined only to the tapetal cell layer of the anther (Figure S2). In contrast, the wild‐type ms44 protein fusion was found mainly in the locule. This result indicated that in the absence of proper protein processing through cleavage of the secretory signal peptide, secretion of the Ms44 protein into the locule was blocked.

### *Ms44* plants increase N partitioning from tassel to ear and improve ear development

**Figure 3:**
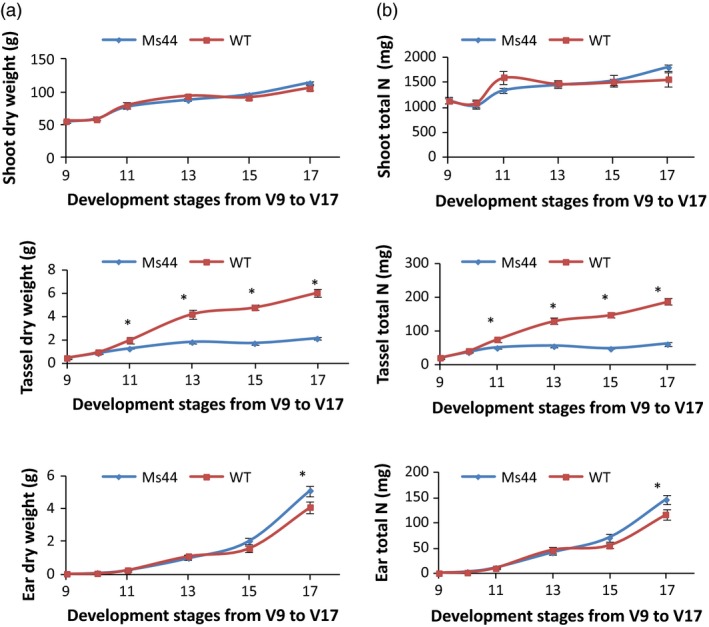
Effect of *Ms44* male sterility on growth and total N content at early reproductive stages. (a) Biomass accumulation of shoot, tassel and ear in *Ms44* male‐sterile plant (Ms44) relative to wild‐type fertile plant (WT) at early reproductive stages from V9 to V17. Tassel is visible fully at V17. Ear weight includes husk. Data are mean ± SEM; five plants were sampled from eight replicated plots for each data point. (b) Total N content of shoot, tassel and ear in *Ms44* male‐sterile plant relative to wild‐type fertile plant at early reproductive stages from V9 to V17. Data are mean ± SEM; five plants were sampled from eight replicated plots for each data point. * indicates a significant difference at *P* < 0.01.

**Figure 4:**
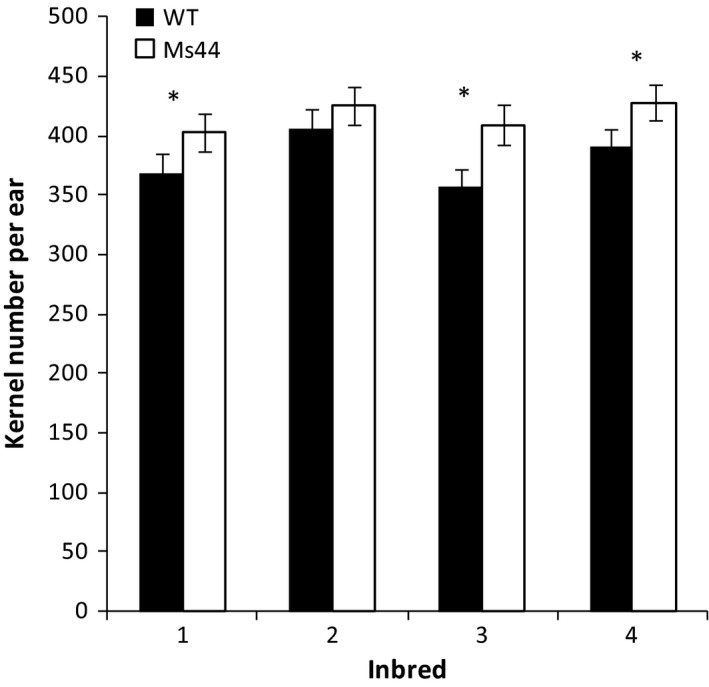
Kernel number per ear of *Ms44* inbred relative to wild‐type inbred. Ears harvested from male‐sterile plants and fertile wild‐type plants were digitally imaged to determine kernel number. * indicates that the kernel number per ear from the *Ms44* sterile inbred was significantly greater than the wild‐type inbred at the *P *< 0.05 confidence level.

Tassel competes with ear for nutrients during early reproductive stages. To determine whether *Ms44* dominant male sterility reduces competition between tassel and ear growth, we measured shoot, tassel, ear biomass and total N content at early reproductive stages from V9 to V17. Shoots from *Ms44* sterile plants did not show a significant difference in biomass or total N content compared to wild type, but the male sterility reduced the accumulation of biomass and N in tassels with an increase in ear biomass and total ear N (Table S1; Figure 3). At V17, male‐sterile plants showed a 64.7% reduction in tassel biomass with a 24.6% increase in ear biomass (Figure 3). More N shifted from tassel to ear growth in sterile plants compared to wild type. As a result, *Ms44* sterile plants produced an average of 9.6% more kernels per ear (35 kernels ear^−1^) than wild‐type plants (Figure 4).

### *Ms44* male‐sterile hybrids increase grain yield under low‐N conditions

To determine whether an increase in kernel number by *Ms44* male sterility translated into higher grain yield, four *Ms44* elite male‐sterile inbreds were each pollinated by 4–5 male testers to produce hybrid seeds for yield trials. Hybrid seed harvested from the heterozygous *Ms44* female plants segregated 50% fertile and 50% male‐sterile (named *Ms44* hybrid). Hybrid seed harvested from wild‐type female plants were 100% fertile and were used as control. We conducted field trials in multiple locations to determine whether the *Ms44* hybrid resulted in a yield advantage compared to fertile sibling controls under limited N, drought and optimal growth conditions. When N was limiting, *Ms44* hybrids increased yield by an average of 4% (352 kg ha^−1^), ranging from 3.3% to 5.7% in 17 hybrids tested under low‐N conditions, and an average of 8.5% (579 kg ha^−1^), ranging from 6.7% to 10.6% in 10 hybrids tested under ultralow‐N conditions, compared to the fertile control. All hybrids showed higher yield increases under extreme low‐N conditions compared to mild‐N stress conditions (Tables 1, S2). When water was limiting during the grain filling period, *Ms44* hybrids increased yield significantly by an average of 1.6% (126 kg ha^−1^). *Ms44*, in three of 17 hybrids, showed a significant increase in yield but did not show a significant yield increase across all 17 hybrids under drought stress during the flowering period (Tables 2, S2). Under optimal conditions, *Ms44* hybrids increased grain yield significantly by an average of 0.9% (126 kg ha^−1^; Tables 1, S2). *Ms44* hybrids showed no yield penalty in any of the tested environments, which is desirable for commercially viable products.

**Table 1:** Grain yield of *Ms44* hybrid and wild type under optimal growth conditions and nitrogen‐limited conditions

Hybrid	Optimal growth conditions				Low‐nitrogen conditions				Ultralow‐nitrogen conditions			
	Isogenic hybrid yield prediction Mg/ha	Ms44 hybrid yield prediction Mg/ha	% change	*N*	Isogenic hybrid yield prediction Mg/ha	Ms44 hybrid yield prediction Mg/ha	% change	*N*	Isogenic hybrid yield prediction 1 Mg/ha	Ms44 hybrid yield prediction Mg/ha	% change	*N*
1	14.09 ± 0.19	14.23 ± 0.19	1.0^a^	33	9.27 ± 0.24	9.68 ± 0.24	4.4^b^	15	6.69 ± 0.31	7.20 ± 0.31	7.7^b^	10
2	14.13 ± 0.18	14.29 ± 0.18	1.1^a^	25	9.13 ± 0.19	9.52 ± 0.19	4.3^b^	24	6.97 ± 0.32	7.54 ± 0.32	8.2^b^	10
3	14.42 ± 0.19	14.54 ± 0.19	0.8	19	10.03 ± 0.22	10.38 ± 0.22	3.5^b^	19	8.10 ± 0.31	8.64 ± 0.31	6.7^b^	10
4	14.21 ± 0.19	14.30 ± 0.19	0.6	20	9.79 ± 0.22	10.16 ± 0.22	3.8^b^	19	7.34 ± 0.32	8.01 ± 0.32	9.2^b^	10
5	14.21 ± 0.19	14.35 ± 0.19	1.0	19	9.49 ± 0.22	9.82 ± 0.22	3.5^b^	20	7.35 ± 0.31	7.89 ± 0.31	7.4^b^	10
6	13.96 ± 0.18	14.15 ± 0.18	1.4^a^	24	8.76 ± 0.19	9.12 ± 0.19	4.1^b^	24	6.63 ± 0.31	7.33 ± 0.31	10.7^b^	10
7	13.76 ± 0.19	13.86 ± 0.19	0.7	36	8.94 ± 0.24	9.24 ± 0.24	3.3^b^	14	6.84 ± 0.32	7.40 ± 0.31	8.2^b^	10
8	13.94 ± 0.18	14.05 ± 0.18	0.8	36	8.83 ± 0.24	9.16 ± 0.24	3.7^b^	14	7.30 ± 0.31	7.86 ± 0.31	7.6^b^	10
9	13.93 ± 0.19	14.13 ± 0.19	1.4^a^	18	8.80 ± 0.22	9.20 ± 0.22	45^b^	20	6.52 ± 0.31	7.12 ± 0.31	9.2^b^	10
10	13.56 ± 0.18	13.66 ± 0.18	0.7	24	8.29 ± 0.19	8.59 ± 0.19	3.7^b^	24	6.35 ± 0.31	6.94 ± 0.31	9.3^b^	10
11	13.98 ± 0.19	14.09 ± 0.19	0.7	21	9.47 ± 0.22	9.80 ± 0.22	3.4^b^	19				
12	13.87 ± 0.22	14.03 ± 0.22	1.2^a^	10	8.61 ± 0.24	9.00 ± 0.24	4.4^b^	15				
13	13.41 ± 0.18	13.58 ± 0.18	1.2^a^	23	7.73 ± 0.19	8.17 ± 0.19	5.7^b^	23				
14	13.59 ± 0.21	13.71 ± 0.21	0.9	13	8.10 ± 0.24	8.44 ± 0.24	4.1^b^	16				
15	13.30 ± 0.19	13.40 ± 0.19	0.8	21	8.03 ± 0.22	8.37 ± 0.22	4.2^b^	20				
16	13.89 ± 0.21	14.01 ± 0.21	0.8	11	8.70 ± 0.24	9.03 ± 0.24	3.7^b^	16				
17	13.47 ± 0.19	13.55 ± 0.19	0.6	20	8.27 ± 0.22	8.55 ± 0.22	3.4^b^	19				

Yield trials and statistical analysis were conducted as described in Methods. *Ms44* hybrid was segregating 50% male‐sterile plants and 50% fertile wild‐type plants. Grain yield of *Ms44* hybrid was compared to the wild‐type hybrid with 100% fertile plants. The LN treatment targeted a yield reduction of 30% through N limitation, and the ULN treatment (in which only 10 hybrids were tested) targeted a 50% yield reduction through N limitation. Data shown are the predicted yield ±SE. *N* is the total number of plots tested.

a Significant at *P* < 0.05.

b Significant at *P* < 0.01.

**Table 2:** Grain yield of *Ms44* hybrid and wild type under drought stress conditions

Hybrid	Drought stress during flowering time				Drought stress during grain filling			
	Isogenic hybrid yield prediction Mg/ha	Ms44 hybrid yield prediction Mg/ha	% change	*N*	Isogenic hybrid yield prediction Mg/ha	Ms44 hybrid yield prediction Mg/ha	% change	*N*
1	7.70 ± 0.20	7.92 ± 0.20	2.8^b^	55	8.62 ± 0.27	8.78 ± 0.27	1.9	22
2	7.78 ± 0.25	7.97 ± 0.25	2.4^a^	15	8.92 ± 0.26	9.06 ± 0.26	1.5	19
3	8.25 ± 0.26	8.37 ± 0.27	1.5	9	8.97 ± 0.28	9.08 ± 0.28	1.2	14
4	7.92 ± 0.26	8.03 ± 0.26	1.5	10	8.73 ± 0.28	8.89 ± 0.28	1.8	11
5	7.86 ± 0.26	7.99 ± 0.26	1.6	12	8.81 ± 0.28	9.02 ± 0.28	2.4^a^	14
6	7.55 ± 0.25	7.68 ± 0.25	1.7	14	8.57 ± 0.26	8.73 ± 0.26	1.8	19
7	7.50 ± 0.20	7.58 ± 0.20	1.1	55	8.50 ± 0.27	8.64 ± 0.27	1.7	23
8	7.18 ± 0.20	7.25 ± 0.20	0.9	55	8.24 ± 0.27	8.38 ± 0.27	1.8	23
9	7.51 ± 0.26	7.70 ± 0.26	2.5^a^	12	8.43 ± 0.28	8.58 ± 0.28	1.8	13
10	7.33 ± 0.25	7.45 ± 0.25	1.6	15	8.30 ± 0.26	8.43 ± 0.26	1.6	18
11	7.84 ± 0.26	7.93 ± 0.26	1.1	11	8.41 ± 0.28	8.55 ± 0.28	1.7	14
12	7.55 ± 0.28	7.71 ± 0.28	2.1	6	8.60 ± 0.30	8.70 ± 0.30	1.2	10
13	6.73 ± 0.25	6.86 ± 0.25	2.0	15	8.10 ± 0.26	8.25 ± 0.26	1.8	17
14	6.92 ± 0.28	7.04 ± 0.28	1.7	6	8.19 ± 0.30	8.30 ± 0.30	1.4	10
15	6.66 ± 0.26	6.76 ± 0.26	1.5	11	7.67 ± 0.28	7.82 ± 0.28	2.0	14
16	7.50 ± 0.28	7.58 ± 0.28	1.2	7	8.49 ± 0.30	8.59 ± 0.30	1.2	8
17	6.83 ± 0.26	6.88 ± 0.26	0.7	12	7.96 ± 0.28	8.02 ± 0.28	0.7	15

Yield trials, drought treatment and statistical analysis were conducted as described in Methods. *Ms44* hybrid was segregating 50% male‐sterile plants and 50% fertile wild‐type plants. Grain yield of *Ms44* hybrid was compared to the wild‐type hybrid with 100% fertile plants. Data shown are the predicted yield ±SE. *N* is the total number of plots tested.

a Significant at *P* < 0.05.

b Significant at *P* < 0.01.

### Transgenic *Ms44* maintainer line development

**Figure 5:**
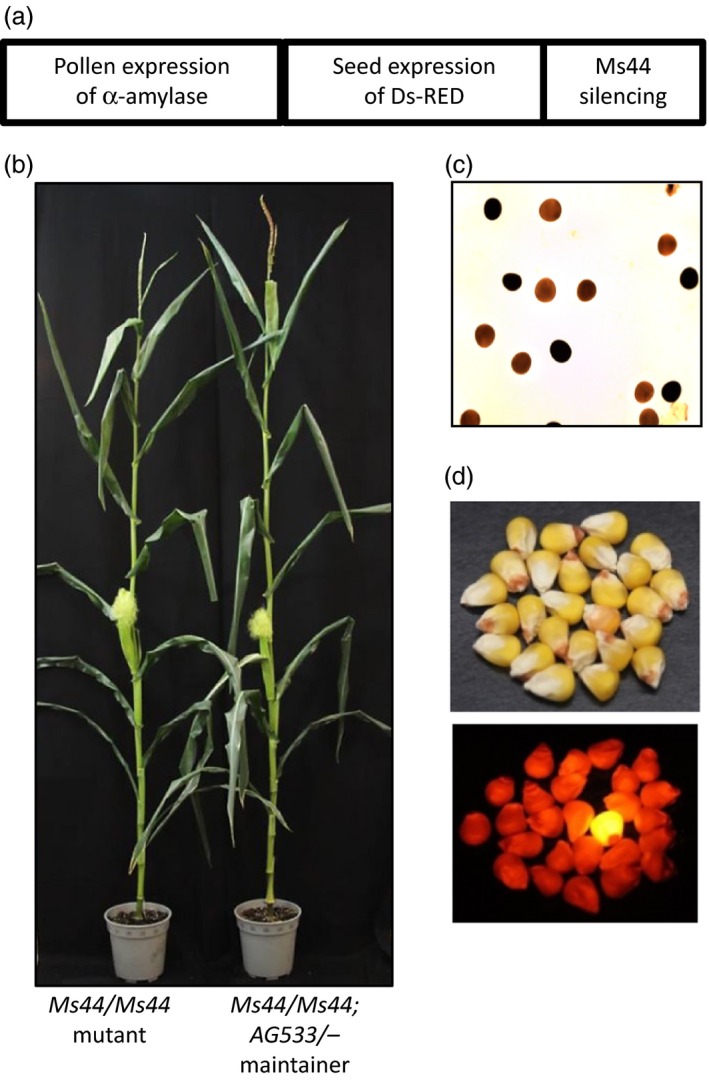
Development of transgenic *Ms44* maintainer line. (a) *AG533* construct map. The Maintainer construct containing three expression cassettes was transformed into a male‐sterile inbred which was heterozygous for *Ms44*. (b) *Ms44* homozygous mutant plant (left) and maintainer plant (right) with fertility restored by expressing *AG533*. (c) Transgenic pollen fertility ablated by overexpression of α‐amylase in pollen. Pollen grains were stained for starch granules using potassium iodide solution (I2KI). The transgenic pollen containing *AG533* were not viable for pollination due to starch breakdown by α‐amylase (brown stained pollen). The wild‐type pollen (dark stained) were viable for pollinating male‐sterile inbred. (d) Expression of DsRed2 in seed as a marker for seed sorting. Transgenic maintainer line seed is red, while nontransgenic seeds are yellow (lower panel is under fluorescence light).

Dominant male‐sterile mutants such as *Ms44* pose additional challenges for seed propagation as the plants carrying the *Ms44* mutation cannot be self‐pollinated. Given that *Ms44* male‐sterile hybrids outperform their fertile siblings under N‐limited conditions, we have modified the previously described seed production technology (SPT; Wu *et al*., 2015) to create a transgenic maintainer line. Instead of complementing a recessive male‐sterile mutation with a wild‐type allele, *Ms44* fertility can be restored by silencing *Ms44* expression using an artificial miRNA (amiRNA). The pollen nonviability and seed colour marker cassettes in the maintainer construct (*AG533*) were identical to the previously described SPT (Figure 5a). The *AG533* construct was transformed into a heterozygous *Ms44* inbred line. As expected, silencing the expression of *Ms44* in mutant plants fully restored male fertility (Figure 5b). Because the amiRNA was directed against the coding sequence of *Ms44*, both the dominant sterility allele and the wild‐type allele were targeted and silenced. Interestingly, the wild‐type plants containing the *AG533* construct remained fertile, suggesting that the *ms44* gene is not absolutely required for male fertility. Expression of α‐amylase using the pollen‐specific *PG47* promoter reduced starch content in the transgenic pollen grains and resulted in those being nonviable (Figure 5c). Seeds expressing red fluorescent protein using an aleurone‐specific Ltp2 promoter were visually distinguishable from nontransgenic yellow seed under appropriate illumination (Figure 5d). Lines with the *AG533* insertion segregating independently from the native *Ms44* locus were selected for advancement. T1 transgenic lines (*Ms44*/−; *AG533*/−) were self‐pollinated to obtain *Ms44* homozygous mutant (*Ms44/Ms44*; −/−) and sibling maintainer lines (*Ms44/Ms44*;* AG533*/−). *Ms44* homozygous mutant plants clearly showed larger ears with more silks and were shorter when compared to the fertile maintainer plants, but ear height was similar between the two (Figure 5b). For the maintainer line, the *Ms44* allele was present in all pollen, with half of that harbouring the unlinked *AG533* construct. Because *AG533* pollen grains were not viable due to α‐amylase activity (Figure 5c), the transmission of the *AG533* construct occurred only through the female gametes. Thus, crossing the homozygous *Ms44* male‐sterile inbred with pollen from the maintainer resulted in only nontransgenic yellow seed progeny.

In practice, the maintainer line can be increased by self‐pollination followed by the selection of red seeds (Figure S3a). The homozygous *Ms44* male‐sterile inbred can be increased with pollen from the sibling maintainer. The progeny seed should be yellow in colour and not contain the transgenic *AG533* construct (Figure S3b). In hybrid seed production, the female inbred is completely male‐sterile (*Ms44/Ms44*), and any cross with a male parent (*ms44/ms44*) will result in F1 hybrid seed that also will be male‐sterile (*Ms44/*−) due to dominance. To enable adequate pollination in the grower's field, blending of the male‐sterile hybrid with a male fertile hybrid will be required at a certain percentage. This can be accomplished by adding a step after the initial maintainer line cross during inbred increase. Pollination of the male‐sterile *Ms44* homozygous inbred with the wild‐type inbred will generate heterozygous *Ms44* male‐sterile female inbred plants. Subsequent crosses with any male inbred during hybrid production will result in 50% male‐sterile hybrid progeny (Figure S3c).

## Discussion

Plant nonspecific lipid transfer proteins (nsLTPs) are small proteins with eight conserved cysteine residues and unique secondary protein structures. All LTPs have an N‐terminal ER‐targeting signal peptide for processing in the secretory pathway. This family of protein involved in numerous biological processes, such as lipid transfer, pathogen defence, abiotic stress response and anther development (Liu *et al*., 2015). The maize genome contains 63 nsLTP genes which can be divided into five types (Wei and Zhong, 2014). *Ms44* encodes a lipid transfer protein containing eight conserved cysteine residues and has a secondary protein structure that falls within the type III or alternatively named type C (Figure 1; Boutrot *et al*., 2008; Edstam *et al*., 2011; Wei and Zhong, 2014) of nsLTPs, due to its intron position 1 bp past the last conserved cysteine (C8) codon and the presence of twelve amino acid residues between C6 and C7, which is specific for this class. The type III or type C is found only in plants that produce pollen and seeds (Boutrot *et al*., 2008; Edstam *et al*., 2011). LTP transcripts have been shown to be highly abundant in male organs, comprising 8% of the total transcripts found in rice anthers and over 10% of tapetal transcripts in *Arabidopsis*, and may play an important role in anther development (Huang *et al*., 2009). In *Arabidopsis* and rice, type III LTPs have tapetal specific expression coupled with an ER‐targeting signal sequence, and are secreted from the tapetum via the ER–Golgi system and then become components of the microspore exine (Huang *et al*., 2013; Zhang *et al*., 2010). The maize *ms44* gene expresses specifically in tapetal cells. Fusions of the wild‐type ms44 gene with GFP (ms44‐GFP), as shown in Figure 2e, demonstrate that the Ms44 protein is also secreted into the locule via the ER–TGN. In contrast, the *Ms44* mutant protein is confined to tapetal cells and is not secreted into the locules (Figures 2e, S2). A single amino acid change at the signal peptide cleavage site disrupts cleavage of the signal peptide and blocks secretion of Ms44 protein into the locule, resulting in dominant male sterility (Figure 2). Interestingly, the maize wild‐type *ms44* gene is not required for fertility as plants with silencing of the wild‐type *ms44* gene showed normal pollen development. The A9 gene in *Brassica* was shown not to be required for male fertility by an antisense knockdown experiment and no phenotype was found for RNA interference knockdown (RNAi) of individual type III LTPs in *Arabidopsis*, although RNAi targeting two *Arabidopsis* type III LTPs did have some effect on intine morphology but not overall male fertility (Huang *et al*., 2013; Turgut *et al*., 1994). In contrast, silencing of OsC6 reduced pollen fertility in rice (Zhang *et al*., 2010). This may not be surprising given the abundance of anther‐expressed LTPs. Maize has three closely related type III LTP genes (Wei and Zhong, 2014). Clearly, the Ms44 protein itself is not critical for proper pollen development, but the single amino acid change at the secretory signal cleavage site results in dominant male sterility phenotype, indicating that tapetum secretion is a critical step for the dominant phenotype. It would be interesting to determine whether secretion of other proteins from tapetal cells is impeded in the *Ms44* mutant, or whether blocking protein secretion by chemical BFA treatment would also lead to dominant male sterility.

Maize hybrid seed production requires the crossing of two inbred parent lines to produce the F1 hybrid seed sold to a grower. The female inbred parent needs to be prevented from shedding pollen to ensure pure hybrid seed production. Relative to other hybrid seed production systems, *Ms44* technology has four advantages (Chen and Liu, 2014; Kempe and Gils, 2011; Perez‐Prat and van Lookeren Campagne, 2002). First, when compared to detasseling, *Ms44* inbred lines show an average of 9.4% increase (*P *< 0.05) in kernel numbers per ear (34.7 kernels ear^−1^) over wild type (Figure 4). Hybrid seed yield from the female inbred is important for the cost of hybrid seed production. An increase in female inbred yield, especially in the number of kernels, reduces hybrid seed production costs. Although effective, mechanical detasseling is time‐consuming and labour‐intensive and can be imperfect. It also reduces seed yields due to damage to the upper leaves and stalk. Second, compared to other sterility technologies (Feng *et al*., 2014; Weider *et al*., 2009), such as cytoplasmic male sterility (CMS) or Roundup hybridization system (RHS), *Ms44* is a native genetic male‐sterile mutant that has shown to be stable across different germplasms and broad environments. CMS technology is restricted to certain germplasms and may result in poor sterility stability in inbreds or poor fertility restoration in hybrids depending on growth environments. The transgenic RHS is based on tissue specific expression of a 5‐enolpyruvylshikimate 3‐phosphate synthase (EPSPS). Application of glyphosate herbicide at V8‐to‐V13 growth stages prevents pollen development and eliminates the production of viable pollen in the female inbred parent during the hybrid production phase. Large‐scale spraying of chemicals across all production field acreages is required at a narrow developmental window for this technology to be effective. Hybrid production costs increase, and hybrid purity and production itself may be at risk if complete sterility is not achieved in the female parents. This could be a consequence of field variability as well as environmental variability. Third, although *Ms44* inbred seeds are propagated using a transgenic maintainer line, the progeny do not inherit the transgenic insertion; thus, commercial hybrid seeds and commodity grain are nontransgenic, with respect to the maintainer transgenes. Finally, the dominant *Ms44* hybrid production system is an elegant and simple method to produce blends of male‐sterile F1 hybrids. Recessive *ms45* male‐sterile mutant needs to be bred into both the male and female inbred parents to produce sterile hybrid F1 plants, while the dominant *Ms44* only needs to be in the female inbred parent, reducing breeding efforts by half. Another benefit of the *Ms44* dominant system is that sterility is 100% maintained in the hybrid production field even with contamination during inbred increase, whereas, for *ms45* recessive sterility, any contamination in the inbred increase field results in fertile females in the hybrid production field.

Maize hybrid yields have been increased over the past 50 years, in part, by indirectly selecting for reduced tassel size (Duvick and Cassman, 1999). Reduction in tassel dominance through a dominant genetic male sterility mechanism with a concomitant yield increase is consistent with the indirect selection for smaller tassels performed by maize breeders. Multiple studies have indicated that detasseling and cytoplasmic male‐sterile hybrids improved grain yield under higher population densities for some cultivars (Chinwuba *et al*., 1961; Duvick, 1958). We report here that dominant *Ms44* male‐sterile hybrid improved grain yield under low‐N conditions across all 17 hybrids tested. The efficient use of N can be improved either by increasing N uptake and assimilation or by increasing N partitioning to grain production. Numerous studies have shown that manipulation of candidate genes involved in N uptake, assimilation, root development and N signalling improved NUE using transgenic approaches, but none have demonstrated a significant improvement of NUE in field trails in elite germplasm (McAllister *et al*., 2012; Xu *et al*., 2012). The most advanced lead is alanine aminotransferase. Expression of a barley alanine aminotransferase in canola and rice increased biomass and seed yield under low‐N conditions (Good *et al*., 2007; Shrawat *et al*., 2008). To our knowledge, this is the first report demonstrating that male sterility caused by a single point mutation in *Ms44* improves N use for grain production. The blends of *Ms44* male‐sterile F1 hybrids increase maize yield under stress conditions in elite hybrids, especially under nitrogen‐limited conditions across multiple location field trials (Table 1). While *Ms44* male sterility does not change the total N content, the sterility improves N utilization efficiency by reducing the N use in tassel and pollen development and portioning more N to immature ear development, resulting in an increase in kernel number (Figures 3, 4). Maize is the most widely cultivated crop in sub‐Saharan Africa and provides 70% of the total human caloric intake. Almost 80% of African countries are challenged with nitrogen scarcity in the soil with an average maize yield of 1.6 Mg/ha. On average, farmers apply less than 20 kg/ha N fertilizer in Africa (Potter *et al*., 2010; Vitousek *et al*., 2009). *Ms44* has the greatest potential to increase maize grain yield without increasing N application in countries where farmers cannot afford to apply more N fertilizer but more food production is needed (Edmonds *et al*., 2009; Liu *et al*., 2010). Further testing is needed to validate whether *Ms44* increases grain yield in local germplasms in African low‐N soil. In addition, the *Ms44* hybrid seed production system provides a cost‐effective method to produce high‐quality hybrid seed, which can promote the adoption of hybrid maize in this region. Currently, only 20% of the total maize in Africa is hybrid (Gaffney *et al*., 2016). In North America, although farmers apply sufficient amounts of nitrogen, more than 10% of maize fields are still confronted with nitrogen stress due to nitrate leaching and run‐off. Furthermore, N levels can vary significantly across a single field and parts of the field may experience yield loss due to N stress. *Ms44* male‐sterile hybrids provide an insurance towards stabilizing grain yields if nitrogen stresses occur. The successful use of *Ms44* hybrid seed production technology would not only reduce maize hybrid seed production costs and protect yield under low‐nitrogen conditions, but would also provide benefits to the environment resulting from increased nitrogen use efficiency in maize.

## Experimental procedures

### Map‐based cloning and gene characterization

*Ms44*, a dominant male‐sterile mutant, was initially identified from an EMS mutagenized W23 population and mapped to Chr4 (Albertsen and Trimnell, 1992). A map‐based cloning approach was used to identify the gene and mutation responsible for the sterile phenotype. A BC6‐F2 segregating population was generated by backcrossing *Ms44* into B73 and then crossed to Mo17. Single nucleotide polymorphism (SNP) markers were selected across the previously identified region of Chr4, and 414 individuals were used to confirm the *Ms44* interval. Subsequently, 2686 individuals were genotyped and the *Ms44* interval was defined between PHM9699‐11 (~193 701 964 bp, B73Ref_v3) and PHM16132‐4 (~195 800 812 bp, B73Ref_v3), with 39 recombinants within the interval. Additional SNP markers were developed and the Ms44 interval was further delimited between two CAPS markers, 9212_4 (~195 191 010 bp, B73Ref_v3, 9212_4 forward primer CAGTCCTGCTCGGAGCTTGCTT/reverse primer ACCGAAGGATGCCTGGGAAT) and 2221_3 (~195 300 664 bp, B73Ref_v3, 2221_3 forward prime AGTTGTTGTGCTTGAAGTACTTGGG/reverse primer GGTCATAGGCTTTCAAGTGTACACA).

### Vector construction, plant transformation and transgene expression analysis

The *Ms44* (‘MS44DOM’) gene was PCR‐cloned from maize genomic DNA based on the original mapping data. It comprises the 413‐bp coding sequence (two exons interrupted by a 101‐bp intron), 1221 bp of upstream promoter sequence (including the 5′ UTR) and 722 bp of downstream genomic sequence (including the 3′ UTR). The complete gene was ligated into a vector containing *Agrobacterium* T‐DNA RB and LB components, plus a plant selectable marker (CAMV35S PRO::PAT). This vector was mobilized into *Agrobacterium tumefaciens* LBA4404 cells carrying extra copies of several VIR genes (pSB1 (JT reference)) to create plant transformation vector PHP42163 which was introduced into wild‐type maize. The tassel phenotype was recorded at T0 plants in glasshouse.

An artificial microRNA was designed to silence specifically the *Ms44* allele, using the scaffolding of the maize miR396 h pri‐miRNA as template. The endogenous miR396 h targeting sequence was replaced with TCTTATTCCTCTCCCCTCCTG and the endogenous star sequence replaced with CAGGAGGGCAGAGGAATAAGA. The resulting artificial microRNA was synthesized by GenScript and placed under the control of the MS44 DOM promoter and terminator sequences described above. Gateway™ homologous recombination‐based cloning was used to move this expression cassette into a binary transformation vector and stack it with the pollen‐specific α‐amylase cassette and the *DsRed2* colour marker gene previously described (Wu *et al*., 2015). The final vector *AG533* was introduced into *Ms44* heterozygous inbred line to generate maintainer lines.

Maize transformation was carried out as described previously (Unger *et al*., 2001). Plasmid was co‐integrated into a pSB1 vector in *Agrobacterium tumefaciens* strain LBA4404 and then used to transform maize embryos from a proprietary inbred. Multiple lines were generated for each construct. Single‐copy T‐DNA integration lines that expressed the transgene were selected for advancement to glasshouse or field test.

### Fluorescence microscopy, pollen staining and seed sorting

Male spikelets were harvested and anthers were dissected out of the florets and mounted in 1× PBS on glass slides. Light microscopy images were taken with a Leica (Wetzlar, Germany) DMRXA epifluorescence microscope with a mercury light source using fluorescence, bright‐field and differential interference contrast (DIC) optics. The fluorescent filter sets used were from Chroma Technology (Bellows Falls, VT): Alexa 488 #MF‐105 (exc. 486–500, dichroic 505LP, em. 510–530), DAPI #31013 (exc. 360–370, dichroic 380LP, em. 435–485) and Cy3 #C‐106250 (exc. 541–551, dichroic 560LP, em. 565–605). Images were captured with a Hamamatsu (Hamamatsu City, Japan) ORCA‐Flash4.0 LT digital CMOS camera and images manipulated by Molecular Devices (Downingtown, PA) MetaMorph imaging software. Confocal images were taken with the Leica (Wetzlar, Germany) TCS SPE using the solid‐state 405‐nm laser line with the DAPI setting (exc. 350 and em. 461) and the 488‐nm laser with the GFP setting (exc. 489 and em. 508) using the Leica LAS X software.

Pollen staining and seed sorting were carried out as described previously (Wu *et al*., 2015).

### Materials, yield trials, field experiment design and statistical analysis

The dominant male‐sterile allele *Ms44* was backcrossed into four elite inbred maize lines. Two were dose 6 (BC5) and two were dose 4 (BC3) for the numbers of crosses to the recurrent parent. Four to five ear sources were selected for increase based upon positive marker calls for a donor insertion site, decreased insertion size and overall recurrent parent percentage. We harvested dose 5 (BC4) and dose 7 (BC6) inbred seed respectively to be used for trial hybrid production. These inbred lines segregated 1 : 1 for male sterility and male fertility. Molecular markers were utilized to assure genetic purity of the final inbred lines. It has approached 97% for the dose 5 seed and ~99% for the dose 7. The four converted *Ms44* female inbred parental lines were crossed to 4–5 fertile male inbred parental lines to produce 17 hybrids. The resulting F1 hybrid seed segregated 1 : 1 for male sterility and was used for yield trials. Male fertile sibs of female inbred lines were crossed to the same male inbred lines and used as 100% fertile controls to compare to *Ms44* hybrids segregating 1 : 1 for male sterility in yield trials.

During 2014, *Ms44* hybrids, segregating 1 : 1 for male sterility, were submitted to yield trials in a number of North American locations in which stress conditions (drought and reduced nitrogen fertility) were imposed. The experiment was planted at six optimal growth locations (two replications at each location) where N was applied at rates greater than 224 kg ha^−1^ and water was not limiting (Marion, IA; San Jose, IL; Sciota, IL; York, NE; and a second location in York, NE), at four low‐N locations (four replications at each location) where N was applied at rates ranging from 45 to 78 kg/ha to target yield reduction by approximately 30% but water was not limiting (Johnston, IA; Marion, IA; Sciota, IL; and Woodland, CA), at two flowering stress locations (four replications per location) where N was not limiting and applied at rates greater than 168 kg ha^−1^ but water was withheld during flowering (Woodland, CA; and Plainview, TX), and at three grain filling stress locations (three replications per location) where N was not limiting and applied at rates greater than 168 kg ha^−1^ but water was withheld during the grain fill period (Fruitland, IA; Garden City, KS; and Plainview, TX). In addition, there were two locations that targeted an ultralow‐nitrogen (ULN) treatment (in which only 10 hybrids were included) that targeted a 50% yield reduction through N limitation. One location with an ULN treatment was Woodland, CA, where 22 kg N ha^−1^ was applied at planting and an additional 45 kg N ha^−1^ in the irrigation water may be applied. At the second ULN location, Fruitland, IA, 112 kg N ha^−1^ was applied prior to planting. This location has a high percentage of sand, is irrigated frequently and has a high propensity for leaching N during the season. All low‐N and ULN locations were on sites previously depleted of nitrogen. Urea–ammonium nitrate (UAN) was used as N fertilizer. At all locations, phosphorus and potassium levels were within the optimal range for maize production based on either soil testing results or application of fertilizer to ensure N was the main nutrient limiting crop yield.

An additional field trial was conducted in South America near Viluco, Chile, during the 2014–2015 growing season. The experiment was grown in a N‐depleted location where N was limiting (0 kg N ha^−1^ applied at planting and 72 kg N ha^−1^ was applied via irrigation water), a location where water was withheld during the flowering period (total N applied during the season was 77 kg N ha^−1^), a location where water was withheld during the grain filling period (total N applied during the season was 69 kg N ha^−1^) and a final location where N and water were both adequate (total N applied during the season was 106 kg N ha^−1^). The soils at the experiment location Viluco, CH, provide a large proportion of required N through mineralization and do not require large inputs of fertilizer N to produce grain yields in excess of 12.5 Mg ha^−1^.

The experimental design was a randomized complete block design in a split‐plot arrangement. The main plot consisted of hybrid pedigree and the subplot was 100% male fertile or *Ms44* hybrid segregating for 50% male‐sterile plants. Experimental units in North American and South American trials were all four rows with 76 cm row spacing and a row length from 4.3 to 5.1 m depending on the testing location. At harvest, a combine was used to collect grain weight and grain moisture data from the centre two rows. Yield was calculated and adjusted to a standard moisture of 155 g kg^−1^.

Each location, or field × irrigation × density × experiment, was classified as optimal growth condition, low‐N condition, ultralow‐N condition, flowering drought stress or grain filling drought stress. In each location, hybrids were randomized in incomplete blocks in the main plots, and sterile plants (100% fertile control and the segregating 50 : 50 treatment) were randomized in the subplots. Yield data were modelled using the ASReml‐R package within R (Butler, 2009; R core Team, 2015). The model can be specified as:

y=t+l+s+l*s+r+h+h*l+h*r+h*s+h*s*l+ξ+η

where *y* denotes yield, *t* denotes location classification, *l* denotes location, *s* denotes sterility, *r* denotes block–location combination, *h* denotes hybrid, *ξ* denotes location‐specific residual errors and *η* denotes location‐specific spatial correlations. Location classification, location, sterility and location x sterility interaction were treated as fixed effects. All the other effects were treated as random effects. The mixed model with spatial adjustment allows the reduction of noise caused by field variability while preserving the genetic signal. Yield for sterility cross hybrids and yield for sterility cross hybrids within each location classification were estimated using best linear unbiased estimator (BLUE). Yield for sterility within hybrid was predicted using best linear unbiased predictor (BLUP), as hybrid effect was treated as random. The BLUPs are not strictly equal to the average of the BLUEs because they have been adjusted to reflect hybrid variation. Differences between the 100% fertile control and the *Ms44* segregating 50 : 50 treatment were considered significant at the 5% confidence level.

### Physiological analysis of *Ms44* male‐sterile plants in field

*Ms44* male‐sterile plants produced by expression of *Ms44* genomic DNA were planted in Johnston field in 2013 to determine whether male sterility affects tassel and ear biomass and N accumulation at early reproductive stages. The experimental design was a multistage complete randomized design; eight plots of sterile and eight plots of fertile treatments were completely randomized within each developmental stage. Five plants were harvested from eight replicates and separated into developing ear, tassel and shoots at V9 (with nine leaves at this stage), V10, V11, V13, V15 and V17 (when tassel is visible fully). Samples were dried at 70 °C for 3 days. Dry weight was recorded. Total N content was determined by the combustion method. Averaged data were modelled using the ASReml‐R package within R (Butler, 2009; R Core Team, 2015). The model can be specified as: y=v+s+v*s+ ε, where y denotes averaged trait, v denotes developmental stage, s denotes sterility and ε denotes residual. Stage, sterility and stage x sterility interaction were treated as fixed effects. Sterility within each stage was estimated using best linear unbiased estimator (BLUE).

Four inbred lines carrying the dominant *Ms44* allele were tested to determine the effect of male sterility on kernel number per ear. In the North American trial, a 100% fertile line and a segregating line for each inbred were grown at five locations (Champaign, IL; Macomb, IL; Miami, MO; Princeton, IN; York, NE) in 2014. In South America near Viluco, Chile, the same four segregating inbred lines were tested at four locations. At physiological maturity, 10 ears were harvested from the tagged sterile plants from the segregating row. Ten ears were harvested from fertile plants in the 100% fertile row as the control. From both the North American and South American experiments, harvested ears were prepared for imaging by removing all dry silks. A digital image was collected of sterile and fertile ears and processed to determine kernel number per ear.

## Conflict of interest

This work was funded by DuPont Pioneer, a for‐profit agricultural technology company, as part of its research and development programme. All authors were DuPont Pioneer employees when contributing to this work. B.S., D.L., M.A., J.S., T.F., A.L. and B.L. are inventors on pending patent applications on this work.

## Supporting information


**Figure S1** Expression pattern of *ms44* gene.
**Figure S2** Localization of Ms44 mutant protein in tapetal cells using confocal microscopy.
**Figure S3** Outline of *Ms44* hybrid seed production technology.
**Table S1** Effect of *Ms44* male sterility on shoot, tassel, and ear biomass and their N content.
**Table S2** Estimated average grain yield of *Ms44* hybrid and wild‐type hybrid across all hybrids and locations.

## References

[pbi12689-bib-0001] Albertsen, M.C. and Trimnell, M. (1992) Linkage between Ms44 and C2. Maize Genet. Coop. Newslett. 66, 49.

[pbi12689-bib-0002] Boutrot, F. , Chantret, N. and Gautier, M.F. (2008) Genome‐wide analysis of the rice and Arabidopsis non‐specific lipid transfer protein (nsLtp) gene families and identification of wheat nsLtp genes by EST data mining. BMC Genom. 9, 86. doi:10.1186/1471‐2164‐9‐86. 10.1186/1471-2164-9-86 PMC2277411 18291034

[pbi12689-bib-0003] Butler, D. (2009) ASReml: Asreml () fits the linear mixed model. R package version 3.0‐1. Hemel Hempstead, UK: VSN International Ltd. http://www.vsni.co.uk.

[pbi12689-bib-0004] Chen, L. and Liu, Y.G. (2014) Male sterility and fertility restoration in crops. Annu. Rev. Plant Biol. 65, 579–606. 24313845 10.1146/annurev-arplant-050213-040119

[pbi12689-bib-0005] Chinwuba, P.M. , Grogan, C.O. and Zuber, M.S. (1961) Interaction of detasseling, sterility, and spacing on yields of maize hybrids. Crop Sci. 1, 279–280.

[pbi12689-bib-0006] Ciampitti, I.A. and Vyn, T.J. (2014) Understanding global and historical nutrient use efficiencies for closing maize yield gaps. Agron. J. 106, 2107–2117.

[pbi12689-bib-0007] Duvick, D.N. (1958) Yields and other agronomic characteristics of cytoplasmically pollen sterile corn hybrids, compared to their normal counterparts. Agron. J. 50, 121–125.

[pbi12689-bib-0008] Duvick, D.N. and Cassman, K.G. (1999) Post‐green revolution trends in yield potential of temperate maize in the North‐Central United States. Crop Sci. 39, 1622–1630.

[pbi12689-bib-0009] Edmonds, D.E. , Abreu, S.L. , West, A. , Caasi, D.R. , Conley, T.O. , Daft, M.C. , Desta, B. *et al* (2009) Cereal nitrogen use efficiency in sub Saharan Africa. J. Plant Nutr. 32, 21070–22122.

[pbi12689-bib-0010] Edstam, M.M. , Viitanen, L. , Salminen, T.A. and Edqvist, J. (2011) Evolutionary history of the non‐specific lipid transfer proteins. Mol. Plant 4, 947–964. 21486996 10.1093/mp/ssr019

[pbi12689-bib-0011] Feng, P.C. , Qi, Y. , Chiu, T. , Stoecker, M.A. , Schuster, C.L. , Johnson, S.C. , Fonseca, A.E. *et al* (2014) Improving hybrid seed production in corn with glyphosate‐mediated male sterility. Pest Manag. Sci. 70, 212–218. 23460547 10.1002/ps.3526

[pbi12689-bib-0012] Gaffney, J. , Anderson, J. , Franks, C. , Collinson, S. , MacRobert, J. , Woldemariam, W. and Albertsen, M. (2016) Robust seed systems, emerging technologies, and hybrid crops for Africa. Glob. Food Security, 9, 36–44.

[pbi12689-bib-0013] Good, A.G. and Beatty, P.H. (2011) Fertilizing nature: a tragedy of excess in the commons. PLoS Biol. 9, e1001124. doi:10.1371/journal.pbio.1001124. 21857803 10.1371/journal.pbio.1001124 PMC3156687

[pbi12689-bib-0014] Good, A.G. , Johnson, S.J. , De Pauw, M. , Carroll, R.T. , Savidov, N. , Vidmar, J. , Lu, Z. *et al* (2007) Engineering nitrogen use efficiency with alanine aminotransferase. Can. J. Bot. 85, 252–262.

[pbi12689-bib-0015] Han, M. , Okamoto, M. , Beatty, P.H. , Rothstein, S.J. and Good, A.G. (2015) The Genetics of nitrogen use efficiency in crop plants. Annu. Rev. Genet. 49, 269–289. 26421509 10.1146/annurev-genet-112414-055037

[pbi12689-bib-0016] Huang, M.D. , Wei, F.J. , Wu, C.C. , Hsing, Y.I. and Huang, A.H. (2009) Analyses of advanced rice anther transcriptomes reveal global tapetum secretory functions and potential proteins for lipid exine formation. Plant Physiol. 149, 694–707. 19091874 10.1104/pp.108.131128 PMC2633857

[pbi12689-bib-0017] Huang, M.D. , Chen, T.L. and Huang, A.H. (2013) Abundant type III lipid transfer proteins in *Arabidopsis* tapetum are secreted to the locule and become a constituent of the pollen exine. Plant Physiol. 163, 1218–1229. 24096413 10.1104/pp.113.225706 PMC3813645

[pbi12689-bib-0018] Kempe, K. and Gils, M. (2011) Pollination control technologies for hybrid breeding. Mol. Breed. 27, 417–437.

[pbi12689-bib-0019] Kempe, K. , Rubtsova, M. and Gils, M. (2014) Split‐gene system for hybrid wheat seed production. Proc. Natl Acad. Sci. USA, 111, 9097–9102. 24821800 10.1073/pnas.1402836111 PMC4078799

[pbi12689-bib-0020] Liu, J. , You, L. , Aminid, M. , Obersteiner, M. , Herreroe, M. , Zehnderf, A.J.B. and Yang, H. (2010) A high‐resolution assessment on global nitrogen flows in cropland. Proc. Natl Acad. Sci. USA, 107, 8035–8045. 20385803 10.1073/pnas.0913658107 PMC2867927

[pbi12689-bib-0021] Liu, X. , Zhang, Y. , Han, W. , Tang, A. , Shen, J. , Cui, Z. , Vitousek, P. *et al* (2013) Enhanced nitrogen deposition over China. Nature, 494, 459–463. 23426264 10.1038/nature11917

[pbi12689-bib-0022] Liu, F. , Zhang, X. , Lu, C. , Zeng, X. , Li, Y. , Fu, D. and Wu, G. (2015) Non‐specific lipid transfer proteins in plants: presenting new advances and an integrated functional analysis. J. Exp. Bot. 66, 5663–5681. 26139823 10.1093/jxb/erv313

[pbi12689-bib-0023] McAllister, C.H. , Beatty, P.H. and Good, A.G. (2012) Engineering nitrogen use efficiency crop plants: the current status. Plant Biotechnol. J. 10, 1011–1025. 22607381 10.1111/j.1467-7652.2012.00700.x

[pbi12689-bib-0024] Newhouse, K. , Fischer, J. and Gobel, E. (1996) Pollination control with SeedLink™ In 51st Annual Corn and Sorghum Ind. Research Conference (Wilkinson D., ed.), pp. 227–234. Washington, DC: American Seed Trade Association (ASTA).

[pbi12689-bib-0025] Perez‐Prat, E. and van Lookeren Campagne, M.M. (2002) Hybrid seed production and the challenge of propagating male‐sterile plants. Trends Plant Sci. 7, 199–203. 11992824 10.1016/s1360-1385(02)02252-5

[pbi12689-bib-0026] Potter, P. , Ramankutty, N. , Bennett, E.M. and Donner, S.D. (2010) Charactering the spatial patterns of global fertilizer application and manure production. Earth Interact. 14, 1–21.

[pbi12689-bib-0027] R Core Team . (2015) R: A Language and Environment for Statistical Computing. Vienna, Austria: R Foundation for Statistical Computing http://www.R-project.org.

[pbi12689-bib-0028] Raun, W.R. and Johnson, G.V. (1999) Improving nitrogen use efficiency for cereal production. Agronomy J. 91, 357–363.

[pbi12689-bib-0029] Shrawat, A.K. , Carroll, R.T. , DePauw, M. , Taylor, G.J. and Good, A.G. (2008) Genetic engineering of improved nitrogen use efficiency in rice by the tissue‐specific expression of alanine aminotransferase. Plant Biotechnol. J. 6, 722–732. 18510577 10.1111/j.1467-7652.2008.00351.x

[pbi12689-bib-0030] Skibbe, D.S. and Schnable, P.S. (2005) Male sterility in maize. Maydica, 50, 367–376.

[pbi12689-bib-0031] Turgut, K. , Barsby, T. , Craze, M. , Freeman, J. , Hodge, R. , Paul, W. and Scott, R. (1994) The highly expressed tapetum‐specific A9 gene is not required for male fertility in *Brassica napus* . Plant Mol. Biol. 1, 97–104. 10.1007/BF00040577 8111030

[pbi12689-bib-0032] Unger, E. , Betz, S. , Xu, R. and Cigan, A.M. (2001) Selection and orientation of adjacent genes influences DAM‐mediated male sterility in transformed maize. Transgenic Res. 10, 409–422. 11708651 10.1023/a:1012032000383

[pbi12689-bib-0033] Vitousek, P.M. , Naylor, R. , Crews, T. , David, M.B. , Drinkwater, L.E. , Holland, E. , Johnes, P.J. *et al* (2009) Nutrition imbalances in agricultural development. Science, 324, 1519–1520. 19541981 10.1126/science.1170261

[pbi12689-bib-0034] Von Heijne, G. (1986) A new method for predicting signal sequence cleavage sites. Nucleic Acids Res. 11, 4683–4690. 10.1093/nar/14.11.4683 PMC311474 3714490

[pbi12689-bib-0035] Wei, K. and Zhong, X. (2014) Non‐specific lipid transfer proteins in maize. BMC Plant Biol. 14, 281. doi:10.1186/s12870‐014‐028‐8. 25348423 10.1186/s12870-014-0281-8 PMC4226865

[pbi12689-bib-0036] Weider, C. , Stamp, P. , Christovc, N. , Hüskend, A. , Foueillassare, X. , Campb, K.H. and Munsch, M. (2009) Stability of cytoplasmic male sterility in maize under different environmental conditions. Crop Sci. 49, 77–84.

[pbi12689-bib-0037] Wright, S.Y. , Suner, M.M. , Bell, P.B. , Vaudin, M. and Greenland, A.J. (1993) Isolation and characterization of male flower cDNAs from maize. Plant J. 3, 41–49. 8401606 10.1046/j.1365-313x.1993.t01-2-00999.x

[pbi12689-bib-0038] Wu, Y. , Fox, T.W. , Trimnell, M.R. , Wang, L. , Xu, R.J. , Cigan, A.M. , Huffman, G.A. *et al* (2015) Development of a novel recessive genetic male sterility system for hybrid seed production in maize and other cross‐pollinating crops. Plant Biotechnol. J. 14, 1046–1054. 26442654 10.1111/pbi.12477 PMC5057354

[pbi12689-bib-0039] Xu, G. , Fan, X. and Miller, A.J. (2012) Plant nitrogen assimilation and use efficiency. Annu. Rev. Plant Biol. 63, 153–182. 22224450 10.1146/annurev-arplant-042811-105532

[pbi12689-bib-0040] Zhang, D. , Liang, W. , Yin, C. , Zong, J. , Gu, F. and Zhang, D. (2010) OsC6, encoding a lipid transfer protein, is required for postmeiotic anther development in rice. Plant Physiol. 154, 149–162. 20610705 10.1104/pp.110.158865 PMC2938136

