# Supplementary material for: A single point mutation in Ms44 results in dominant male sterility and improves nitrogen use efficiency in maize
Source: Plant Biotechnol J. 2017 Feb 7;15(8):942–52. doi: 10.1111/pbi.12689 (PMC5506649; doi:10.1111/pbi.12689)
Supplement: Supplementary file 1 — Figure S1 Expression pattern of ms44 gene. Figure S2 Localization of Ms44 mutant protein in tapetal cells using confocal microscopy. Figure S3 Outline of Ms44 hybrid seed production technology. Table S1 Effect of Ms44 male sterility on shoot, tassel, and ear biomass and their N content. Table S2 Estimated average grain yield of Ms44 hybrid and wild‐type hybrid across all hybrids and locations. [file PBI-15-942-s001.docx]

**
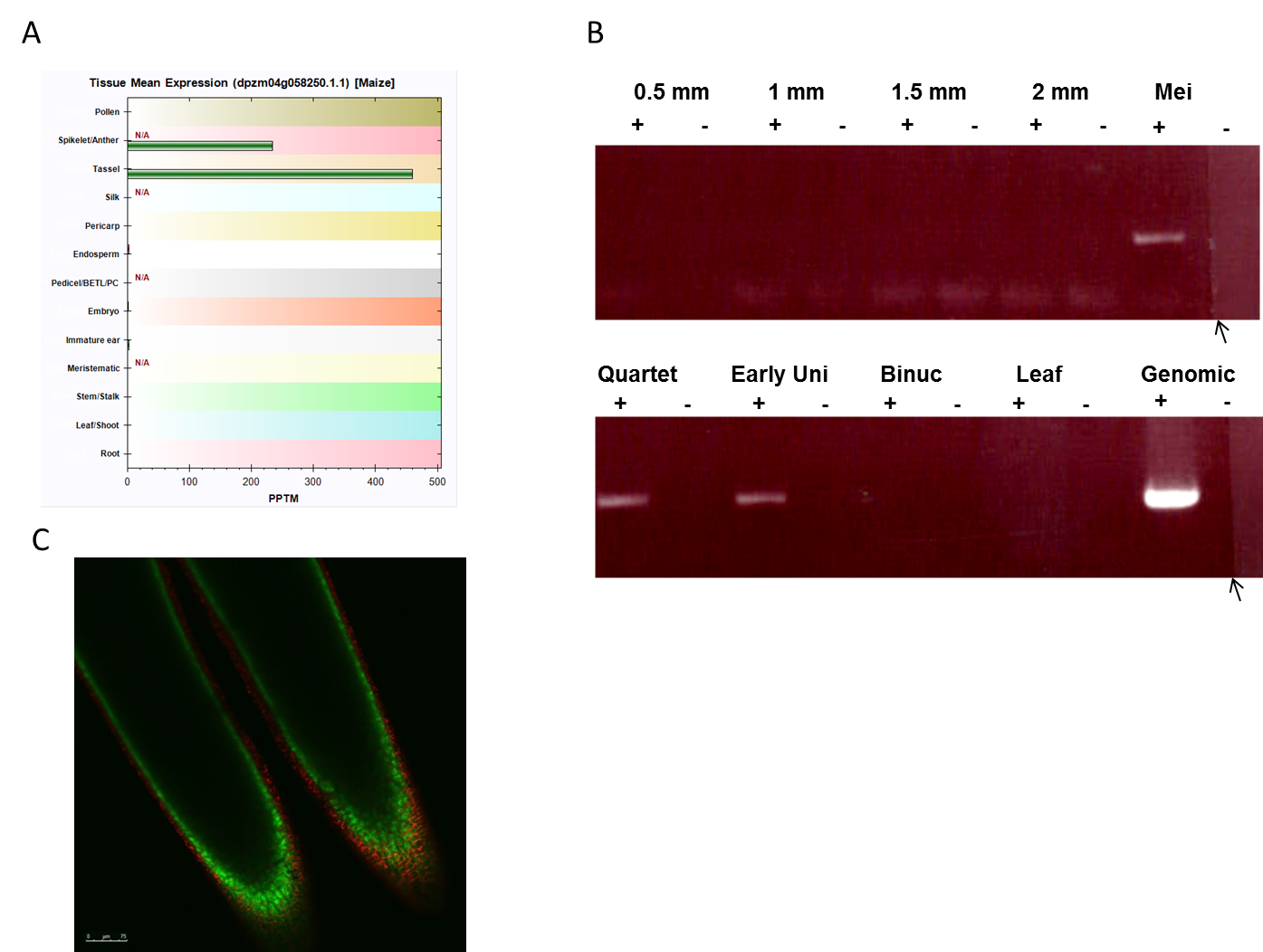
**

**Figure S1** Expression pattern of *ms44* gene. A, Relative *ms44* expression abundance in parts per 10 million in different maize tissues determined by the MPSS technology. B, Temporal expression of ms44 during anther development by RT-PCR . Anther stages prior to meiosis designated by anther size, subsequent stages determined by microscopic microspore evaluation. Each stage run with (+) and without (-) reverse transcriptase. Arrow indicates the edge of gel photo on lab notebook covered by transparent tape. C, Spatial expression of ms44 promoter: Zs-Green fluorescence gene in quartet anthers using confocal microscopy. Red fluorescence is due to auto-fluorescence of chlorophyll in the endothecium anther layer.

Tapetum


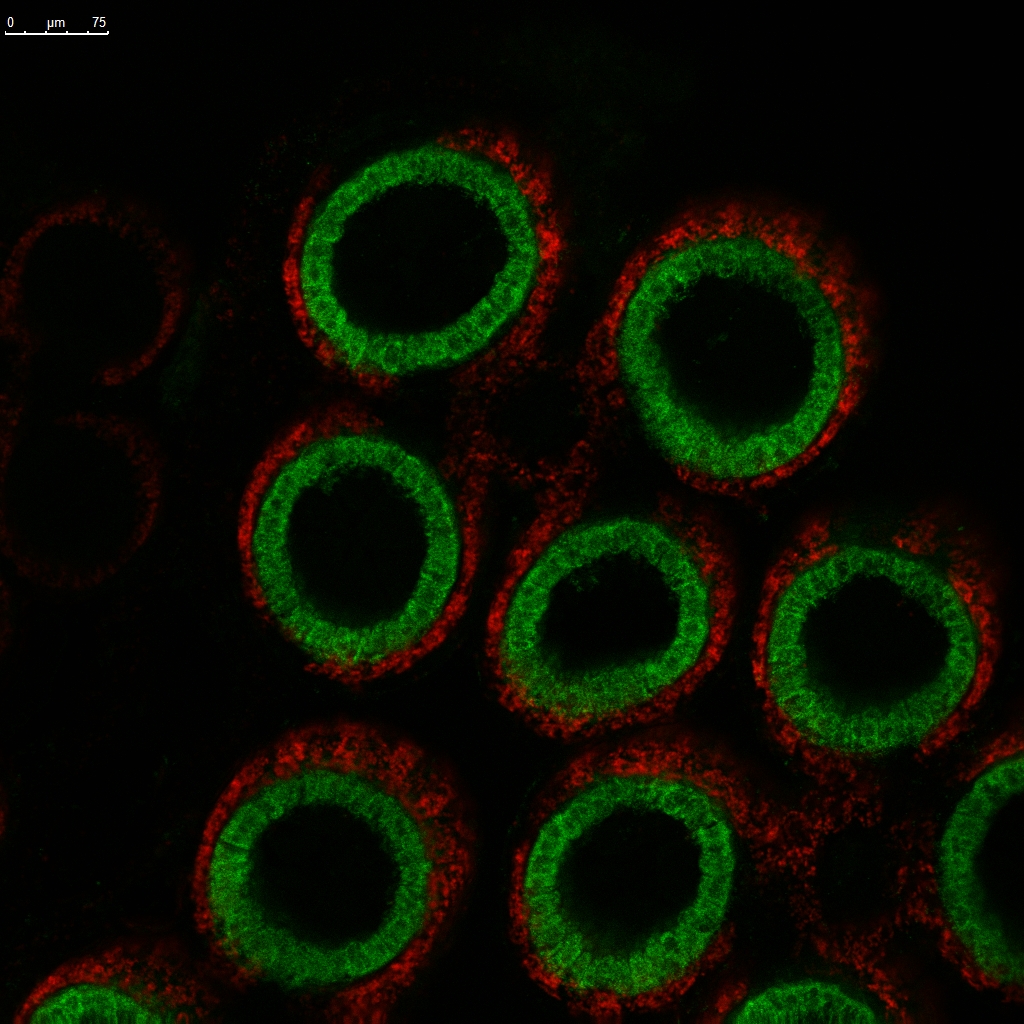

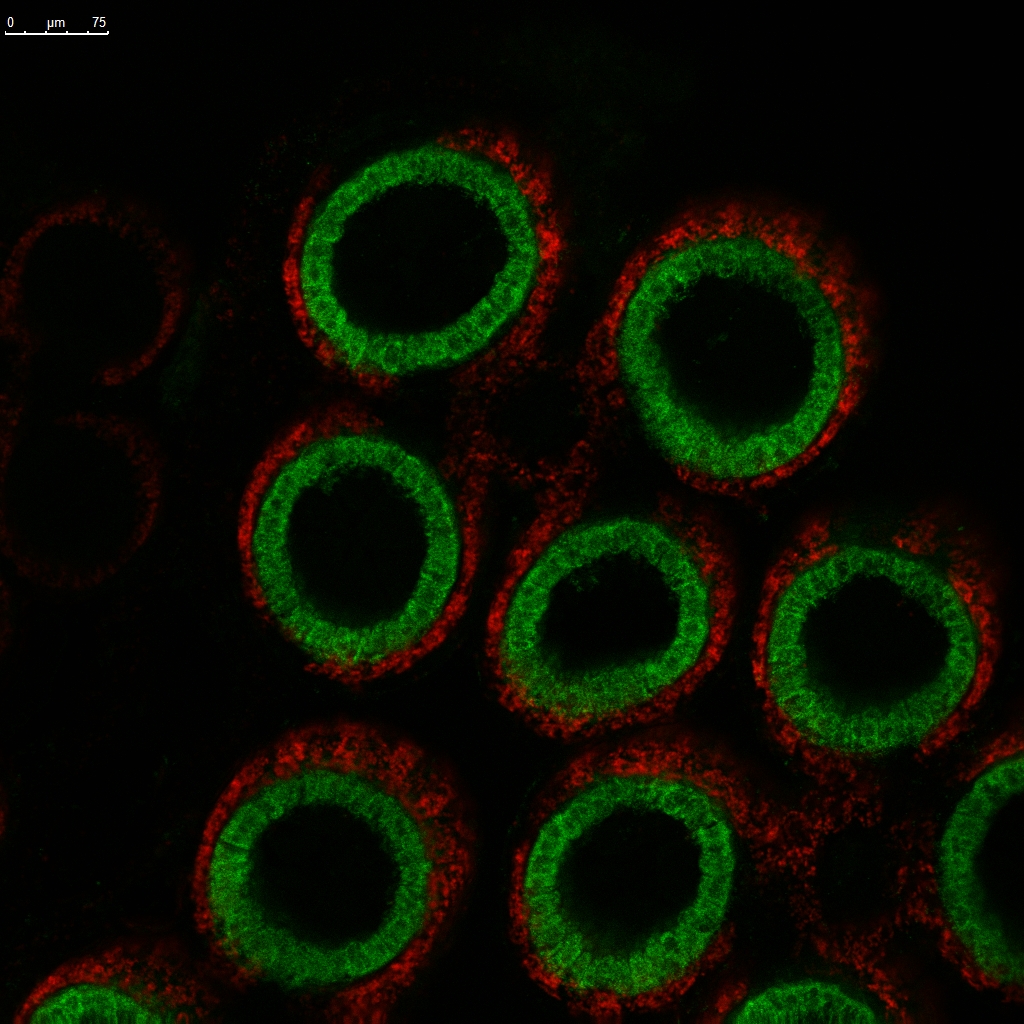


T

**Figure S2** Localization of Ms44 mutant protein in tapetal cells using confocal microscopy. Ms44 mutant protein was fused with AcGFP and expressed in wild type plant under the Ms44 promoter. Ms44 mutant protein-AcGFP was confined to tapetal cells.


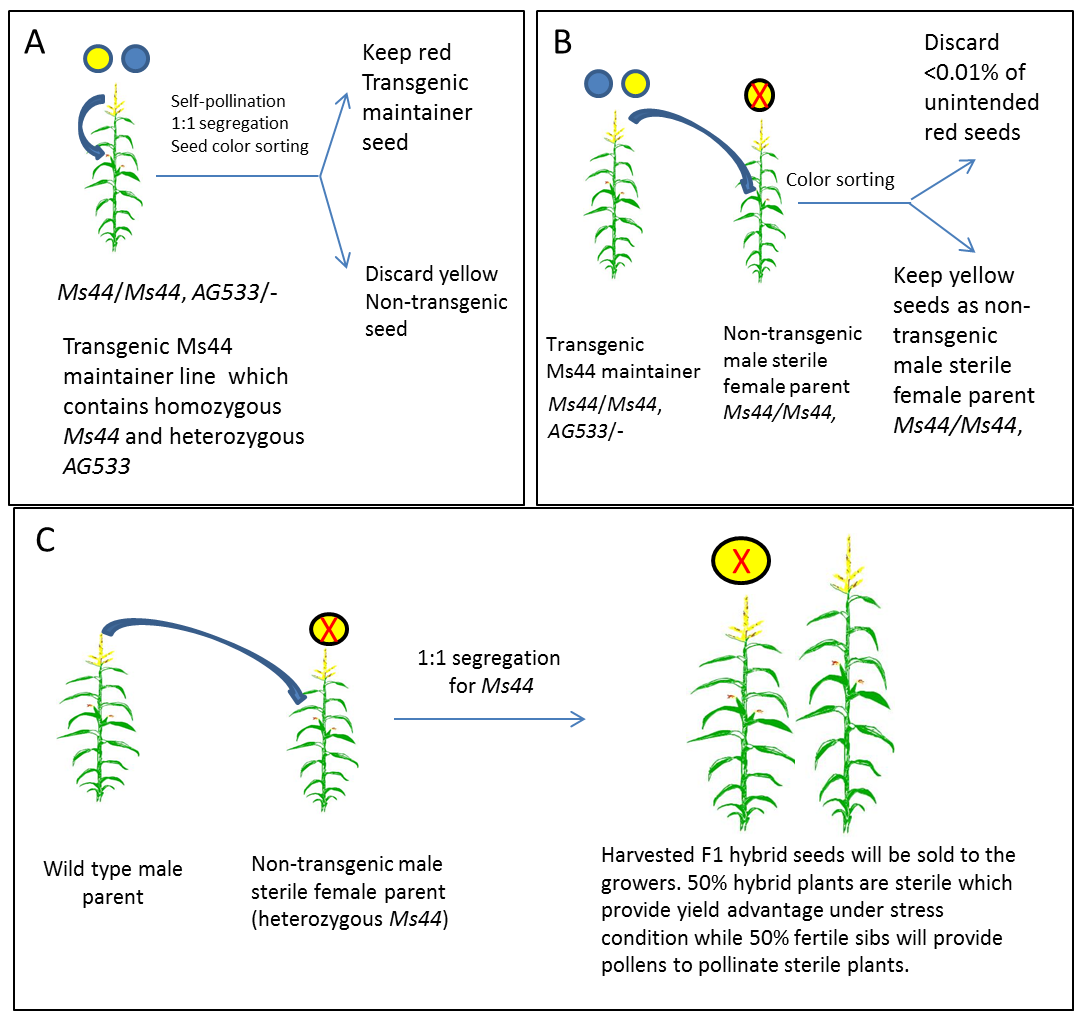


**Figure S3** Outline of *Ms44* hybrid seed production technology. A, Propagation of *Ms44* maintainer line. The maintainer line was increased by self-pollination. Transgenic *Ms44* pollen (blue color) containing *AG533* were not viable and are not expected to be transmitted to the progeny due to starch breakdown by a-amylase. Because the PG47 promoter is exquisitely pollen specific, it does not express in the ear and therefore allows the construct to be transmitted through the female. Self-pollinated seeds were segregating 1:1 for red maintainer seeds and yellow non-transgenic *Ms44* seeds, which can be separated by color sorting. B, Propagation of non-transgenic male sterile female inbred parent line. Non-transgenic male sterile female parent was pollinated by sib maintainer line. The maintainer line shed 50% non-transgenic *Ms44* pollen (yellow color) and 50% transgenic *Ms44* pollen (blue color). Transgenic *Ms44* pollen containing *AG533* were not viable and are not expected to be transmitted to the progeny. C, Production of 50% male sterile blending hybrid seeds. The homozygous *Ms44* plants were pollinated by wild type sib to produce heterozygous *Ms44* seeds. Non-transgenic male sterile *Ms44* heterozygous female inbred were pollinated by a wild type male parent to produce F1 hybrid seeds which segregated 1:1 for *Ms44* and wild type allele.

**Table S1. Effect of *Ms44* male sterility on shoot, tassel, and ear biomass and their N content**

| Trait name | Source | *df*^a^ | *F* value | *P*-value^b^ |  |  |  |  |
| --- | --- | --- | --- | --- | --- | --- | --- | --- |
| Mean.shoot.wt.(g) | stage | 5, 84 | 92.92 | <0.001 |  |  |  |  |
|  | sterility | 1, 84 | 0.2019 | 0.654 |  |  |  |  |
|  | stage x sterility | 5, 84 | 1.259 | 0.289 |  |  |  |  |
| Mean.tassel.wt.(g) | stage | 5, 84 | 131.1 | <0.001 |  |  |  |  |
|  | sterility | 1, 84 | 276.1 | <0.001 |  |  |  |  |
|  | stage x sterility | 5, 84 | 43.27 | <0.001 |  |  |  |  |
| Mean.ear wt.(g) | stage | 5, 84 | 203.3 | <0.001 |  |  |  |  |
|  | sterility | 1, 84 | 5.242 | 0.025 |  |  |  |  |
|  | stage x sterility | 5, 84 | 3.002 | 0.015 |  |  |  |  |
| Shoot total.N.(mg) | stage | 5, 84 | 15.43 | <0.001 |  |  |  |  |
|  | sterility | 1, 84 | 0.01542 | 0.901 |  |  |  |  |
|  | stage x sterility | 5, 84 | 1.716 | 0.140 |  |  |  |  |
| Tassel total.N.(mg) | stage | 5, 84 | 97.28 | <0.001 |  |  |  |  |
|  | sterility | 1, 84 | 293.9 | <0.001 |  |  |  |  |
|  | stage x sterility | 5, 84 | 44.65 | <0.001 |  |  |  |  |
| Ear total.N.(mg) | stage | 5, 84 | 165.1 | <0.001 |  |  |  |  |
|  | sterility | 1, 84 | 5.228 | 0.025 |  |  |  |  |
|  | stage x sterility | 5, 84 | 2.853 | 0.020 |  |  |  |  |
|  |  |  |  |  |  |  |  |  |
| ^a^ *df*, numerator degrees of freedom, denominator degrees of freedom.  ^b^ *F*-test considered significant difference if the *P*-value is less than 0.05. | | | | | | | | |

**Table S2. Estimated average grain yield of Ms44 hybrid and isogenic hybrid across all hybrids and locations**

| Growth condition | Isogenic hybrid yield estimated Mg/ha | Ms44 hybrid yield estimated Mg/ha | Yield % change | N |
| --- | --- | --- | --- | --- |
| Optimal growth condition | 13.87 ± 0.15 | 14.00 ± 0.15 | 0.9** | 373 |
| Low N condition | 8.84 ± 0.18 | 9.19 ± 0.18 | 4.0** | 321 |
| Ultra-low N condition | 6.82 ± 0.30 | 7.40 ± 0.30 | 8.5** | 100 |
| Flowering drought stress | 7.45 ± 0.18 | 7.57 ± 0.18 | 1.7 ^NS^ | 320 |
| Grain filling drought stress | 8.44 ± 0.23 | 8.58 ± 0.23 | 1.6** | 264 |

Yield trials and statistical analysis were conducted as described in Methods. Data shown are the estimated yield ± s.e. N is the total number of plots tested. ** is significant at p<0.01. NS is not significant at p=0.05
